# Towards accurate modeling of noncovalent interactions for protein rigidity analysis

**DOI:** 10.1186/1471-2105-14-S18-S3

**Published:** 2013-11-05

**Authors:** Naomi Fox, Ileana Streinu

**Affiliations:** 1 Department of Computer Science, University of Massachusetts, Amherst, MA, USA; 2 Department of Computer Science, Smith College, Northampton, MA, USA

## Abstract

**Background:**

Protein rigidity analysis is an efficient computational method for extracting flexibility information from static, X-ray crystallography protein data. Atoms and bonds are modeled as a mechanical structure and analyzed with a fast graph-based algorithm, producing a decomposition of the flexible molecule into interconnected rigid clusters. The result depends critically on noncovalent atomic interactions, primarily on how hydrogen bonds and hydrophobic interactions are computed and modeled. Ongoing research points to the stringent need for benchmarking rigidity analysis software systems, towards the goal of increasing their accuracy and validating their results, either against each other and against biologically relevant (functional) parameters. We propose two new methods for modeling hydrogen bonds and hydrophobic interactions that more accurately reflect a mechanical model, without being computationally more intensive. We evaluate them using a novel scoring method, based on the B-cubed score from the information retrieval literature, which measures how well two cluster decompositions match.

**Results:**

To evaluate the modeling accuracy of KINARI, our pebble-game rigidity analysis system, we use a benchmark data set of 20 proteins, each with multiple distinct conformations deposited in the Protein Data Bank. Cluster decompositions for them were previously determined with the RigidFinder method from Gerstein's lab and validated against experimental data. When KINARI's default tuning parameters are used, an improvement of the B-cubed score over a crude baseline is observed in 30% of this data. With our new modeling options, improvements were observed in over 70% of the proteins in this data set. We investigate the sensitivity of the cluster decomposition score with case studies on pyruvate phosphate dikinase and calmodulin.

**Conclusion:**

To substantially improve the accuracy of protein rigidity analysis systems, thorough benchmarking must be performed on all current systems and future extensions. We have measured the gain in performance by comparing different modeling methods for noncovalent interactions. We showed that new criteria for modeling hydrogen bonds and hydrophobic interactions can significantly improve the results. The two new methods proposed here have been implemented and made publicly available in the current version of KINARI (v1.3), together with the benchmarking tools, which can be downloaded from our software's website, http://kinari.cs.umass.edu.

## Introduction

As new generations of bioinformatics systems are released with new features and updated methods, it is vital to ensure that their results continue to match or improve upon previous generations. A number of protein rigidity analysis software systems have been built, including MSU-FIRST (now ProFlex) [1], ASU-FIRST [2], and our own KINARI [3]. All of these take as input a single protein structure in a PDB file and output a decomposition of the protein into rigid clusters. Although all the systems share the same general approach of mechanical modeling and running a pebble-game algorithm, there are substantial variations in both their modeling and in the underlying algorithms.

The main goal in our research is to validate the predictive power of rigidity analysis systems. Towards this goal, we propose new modeling methods for incorporating noncovalent interactions that may improve accuracy. We also propose a general methodology for benchmarking protein rigidity analysis systems. Included in this a method to assign a score to a predicted cluster decomposition, compared with decompositions produced by some other method. This is an adaptation of the B-cubed score from the information retrieval literature, which is used as a comparative score on two clusterings of the same data [4].

We use this evaluation method to benchmark our software, KINARI, against other previously available systems, MSU-FIRST and ASU-FIRST. In our benchmarking we use two data sets: the first is composed of several proteins used to validate the MSU-FIRST software [1,5] and the second is used in the Gerstein Lab to validate the RigidFinder server [6].

*Protein rigidity analysis*. Rigidity analysis is a well-established method, implemented in several software systems, for analyzing flexibility of proteins. With this method, the molecule is modeled as a mechanical framework, in which distance and angle constraints are derived from chemical bonds and other inter-atomic interactions. A fundamental question in protein mechanics is to identify groups of atoms that move rigidly together, called *rigid clusters*, and the inter-connectivities that hold the protein together while permitting flexing. The pebble games are a family of combinatorial algorithms that work correctly and efficiently on certain families of mechanical structures. They has been implemented in several software packages [1-3] and applied to a variety of protein studies, including identification of folding cores [7], dilution analysis [7], protein motion [8], and improved flexibility simulations [9].

Prior to running the algorithm, the molecule is abstracted as a mechanical model, where its stabilizing interactions (covalent bonds, hydrogen bonds, hydrophobic interactions, etc.) induce length or angle constraints between the constituent elements. The constraints eliminate degrees of freedom and their cumulative effect leads to the creation of rigid clusters, which are then identified by graph-based pebble game algorithms.

*Previous approaches*. In previous work, hydrogen bonds (H-bonds) were modeled as mechanically equivalent to covalent bonds, fixing bond length and bond angles at incident atoms [1-3,10]. It has been observed early on that such a method may lead to inaccurate results, such as an almost complete rigidification of the protein model. Since it is known that not all H-bonds have the same strength, an energy function was applied to prune the weakest bonds and exclude them from the model [1]. A universal H-bond energy cutoff, which would produce biologically credible results for any protein input, has never been found. Wells *et al*. [11] point out the discrepancies of the H-bond energy cutoffs in a number of previous studies in the literature.

Also, in previous work, hydrophobic interactions were identified with heuristic approaches [2], and, unlike H-bonds, they had no associated energies. It has been observed that the tuning of the hydrophobic interactions can be just as important as for H-bonds. Gohlke *et al*. [10] comment, in their study of flexibility changes during Ras-Raf complex formation, "Finding the appropriate balance between these interactions [H-bonds and hydrophobics] is thus crucial for an accurate representation of the flexibility characteristics of proteins".

**Figure 1:**
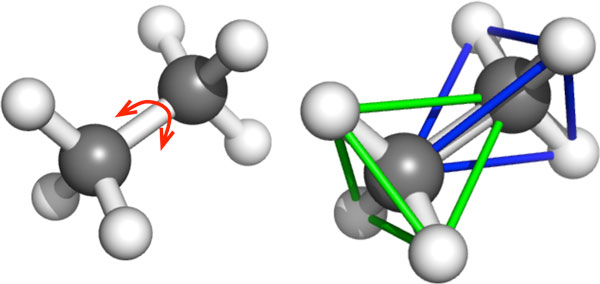
**Body-bar-hinge modeling of molecules**. KINARI builds a body-bar-hinge mechanical model of the input molecule. For example, in ethane, each C atom bonded to four neighbors forms a rigid body. The two bodies share a hinge along the center C-C bond.

*Strength and geometries of H-bonds and hydrophobic interactions*. For covalent bonds, energy and geometry is characterized by the identities of the two electron-sharing atoms. The *bond length *and *directionality *(or *bond-angle*) tend to be fully determined, as explained by molecular orbital theory. For example, in ethane C_2_H_6_, each C atom is bonded to another C atom and 3 H atoms, forming two overlapping, rigid tetrahedra (Figure 1). The bond angles and bond lengths remain relatively fixed. In mechanical modeling for rigidity analysis, covalent bonds are incorporated as bond-length and bond-angle fixing constraints. By contrast, H-bonds display large variations in energies and geometries, even for those with the same donor and acceptor atoms [12] (page 1, paragraph 2). A strong H-bond behaves essentially as a covalent bond, but weaker H-bonds behave more like electrostatic interactions which have much more variance in length and directionality [12]. Pairs of atoms that are packed closely together engage in hydrophobic, or van der Waals, interactions. The strength of these interactions depends on the atom types and pairwise distances. Which H-bonds and hydrophobic interactions to incorporate, and how to model them, is crucial to obtaining accurate results in rigidity analysis.

*Summary of our contributions*. We propose two new methods for incorporating noncovalent interactions for protein rigidity analysis. First, rather than simply removing weaker H-bonds, we propose varying the way that the H-bonds are modeled, based on their strength. In this paper, we investigate modeling the weak H-bonds as a rigid bar which fixes the distance between the endpoints, but permits full rotational freedom. We reveal the limitations of the current mathematical theory for supporting this modeling, and propose heuristics to approximate the rigidity results. The second method we propose is in the inclusion of hydrophobic interactions. We calculate these interactions and assign to them an energy using the Lennard-Jones potential. Then, as for H-bonds, we use an energy cutoff to determine which interactions to include in the modeling. As a proof-of-concept, we investigated the use of a single, rigid bar to model these interactions. We have implemented these extensions in our KINARI software, and made it available for public use on the KINARI-Web server [3]. In addition, we propose a method for evaluating the tuning of the H-bond and hydrophobic energy cutoffs. This is an adaptation of the B-cubed score from the information retrieval literature, which is used to compare two clusterings of the same data [4]. We perform an evaluation on a curated 'gold standard' data set of proteins whose cluster decompositions were computed by a different method. We make the benchmarking scripts, written in python, available at the KINARI web site for public use.

## Background and literature review

We present relevant background material on hydrogen bonds, hydrophobic interactions, rigidity theory, and KINARI's mechanical modeling core. We then provide our literature review.

### Hydrogen bonds in proteins

**Figure 2:**
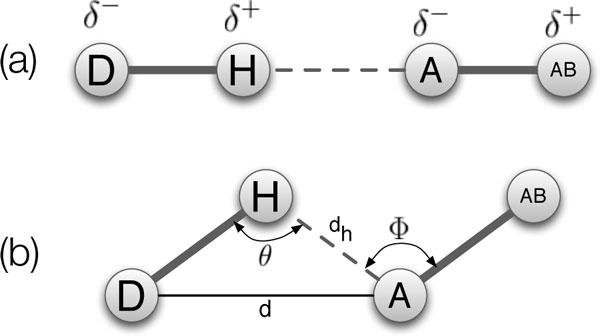
**Hydrogen bond definition**. Hydrogen bond definition. (a) A hydrogen bond forms between an electronegative acceptor atom, A, and a hydrogen atom, H, that is covalently bonded to an electronegative donor atom, D. AB is the acceptor base. (b) Hydrogen bonds are calculated using geometric parameters.

A *hydrogen bond *(H-bond) forms between an electronegative *acceptor *(*A*) atom and a hydrogen atom (*H*) that is covalently bonded to an electronegative *donor *(*D*) atom (Figure 2) [13]. Schematically, we refer to the donor-hydrogen-acceptor triplet as *D*-*H*-*A*.

Secondary structure elements in proteins, mainly alpha helices and beta sheets, are held together by very regular H-bonding patterns along the backbone. H-bonds also form outside secondary structures, helping to interlace the secondary structures or other pieces of the protein together into the folded shape. Intermolecular H-bonds, such as those between two proteins in a complex, or between a protein and a non-protein ligand, play an important role in stabilizing the complex [10,14,15].

*Strong, moderate, and weak H-bonds*. Energies for H-bonds found in proteins are typically under 15 kcal/mol [12] (pg 31-32, Sec. 2.4.2), significantly weaker than covalent bonds with energies around 85 kcal/mol [16]. Weak H-bonds are electrostatic in nature but increasingly behave like covalent bonds as their strength grows [12]. The boundary between 'weak' and 'strong' is blurred. For the modeling scheme proposed here, the boundary between weak and strong is left to the user to determine, as it is the case in all previously implemented methods.

*H-bond energy functions*. H-bonds display large variations in energies and geometries, even for those with the same donor and acceptor atoms. This leads to difficulties in identifying bonds and their strengths, leading to what Gilli and Gilli [12] called the *Hydrogen bond problem*. Despite the difficulty, efforts have been made to quantify the energy, or potential, of individual H-bonds using orientation information alone. The two versions of FIRST and [1,2] and our own KINARI software use the Mayo Lab's energy function [17] for this purpose. This is a closed-form equation, parameterized on H-bond angles and distances, as in Figure 2. The energy function of Kortemme *et al*. is similarly parameterized by angles and distances, but rather than a closed-form equation, it sums the independent energetic contributions from a database of statistics from crystallography-determined protein structures [18].

**Figure 3:**
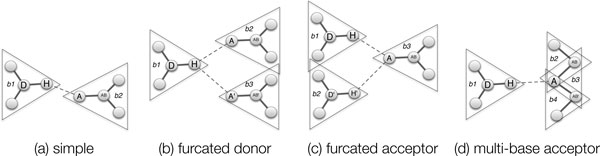
**Hydrogen bond configurations**. Configurations of interest when building a mechanical model of the protein. The triangles show the bodies determined by KINARI for the mechanical model.

*H-bond configurations*. Gilli and Gilli [12](pg. 24) present a review of a number of different configurations that have been studied in the H-bond literature. Figure 3 shows several of them, which are relevant to our modeling. We use the nomenclature from [14] for describing furcated configurations.

In a *simple, non-furcated *configuration, the hydrogen atom, which is covalently bonded to a donor atom, forms a single H-bond with an acceptor atom, which is also covalently bonded to only one atom, the acceptor base (Figure 3a). A *furcated donor *configuration is formed when a single hydrogen atom engages in 2 or more H-bonds (Figure 3b). An acceptor which engages in multiple H-bonds is in a *furcated acceptor *configuration (Figure 3c). These three types of configurations have been identified and studied in the literature. One additional configuration that we identify here, that is not included in Gilli and Gilli's listing, is the *multiple-base acceptor *configuration where an acceptor that is covalently bonded to more than one atom engages in a H-bond (Figure 3d). Later, in the Methods section, we describe why this special configuration is of concern in mechanical modeling.

The most stable configuration of a H-bond is linear, with *D*-*H*-*A *(*θ *in Figure 2) forming a 180^°^angle, but H-bonds are rarely found to be linear, and the most probable value is 165^° ^[13] (pg. 20). Because of geometric constraints, H-bonds in furcated configurations will tend to deviate even more from being linear.

*Frequency of configurations in PDB data*. Furcated hydrogen bond configurations are not rare. Panigrahi and Desiraju [14] performed a survey on H-bond configurations on a data set of structures of 251 proteinligand complexes, using the HBAT software for determining H-bonds. They found that overall 65% of acceptors and 34% of donors were in furcated configurations. Of the furcated acceptor configurations, 66% were bifurcated, 25% were trifurcated, and the remainder engaged in 4 to 6 H-bonds. Of the furcated donor configurations, 39% were bifurcated, 27% were trifurcated, and the remainder were tetrafurcated, pentafurcated, or hexafurcated.

Because KINARI uses HBPLUS (rather than HBAT), the H-bonds identified will differ. Because of this, and because the classification of Panigrahi and Desiraju did not not contain multi-base acceptor configurations, we performed our own examination of configuration frequencies. We examine the 3 proteins from [15] which probed the contribution of non-conventional H-bonds to the rigidity of protein complexes: HIV-1 protease (1htg), serine protease (1vgc), and bilin binding protein (1bbp). The data are shown in Table 1. Multi-base A configurations were found to be uncommon, occurring in approximately 6% of H-bonds.

**Table 1:** Prevalence of H-bonds in special configurations.

	1htg	1vgc	1bbp
total	146	173	553

bifurcated D	1.3%	2.3%	5.8%

bifurcated A	15%	20%	21%

trifurcated A	0%	1.7%	3.8%

multi-base A	4.8%	6.4%	6.0%

We examined the H-bonds calculated with HBPLUS [36] on 3 example proteins from [15]: HIV-1 protease (1htg), serine protease (1vgc), and bilin binding protein (1bbp). We counted how many H-bonds were found in each configuration type shown in Figure 3, which are of special interest in mechanical modeling.

**Figure 4:**
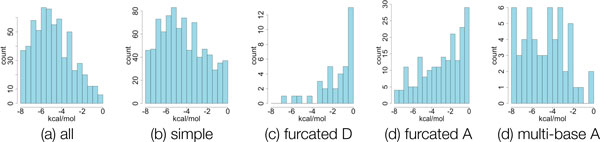
**Histograms of hydrogen bond energies, by configuration**. The distributions of H-bond energies varies based on configuration. H-bonds and associated energies were calculated via HBPLUS [36] software and the Mayo Lab energy function [17] on 3 example proteins from [15]: HIV-1 protease (1htg), serine protease (1vgc), and bilin binding protein (1bbp).

*Configuration energies*. We also calculated the energies of the H-bonds in the 3 proteins, via the Mayo Lab energy function [17]. In Figure 4, we collect together the set of H-bonds from all three proteins, and show the distribution of energies for H-bonds in different configurations. H-bonds in furcated configurations tend to be weaker than non-furcated because the angles tend to deviate further from 180^°^.

### Hydrophobic interactions in proteins

Hydrophobic cores are essential to protein domain stability [16]. These cores are formed from hydrophobic groups concentrating in the center of the protein, minimizing the number of energetically unfavorable contacts with water and maximizing the number of energetically favorable van der Waals interactions. In order to incorporate these interactions into rigidity analysis, the developers of ASU-FIRST introduced a heuristic to to identify and model hydrophobic interactions as pairwise interactions between atoms, that they named 'hydrophobic tethers' [2].

A major limitation of their method was that the identified interactions had no associated energies, and therefore, it was not possible to tune the set of hydrophobic interactions via an energy cutoff. Later, in the Methods section, we will propose another way of calculating and assigning energies to so-called hydrophobic interactions, based on the Lennard Jones potential. A pair of neutral atoms are subject to two distinct forces between them: an attractive force at long ranges (van der Waals force), and a repulsive force at short ranges (Pauli repulsion force). The Lennard-Jones 6-12 potential, shown in the equation below, is an approximation of the sum of these two forces involved in a hydrophobic interaction in proteins. The potential is in standard use in molecular mechanics force fields packages, such as the popular Amber-99 forcefield [19].

(1) $$V=4\varepsilon \left(\frac{\sigma^{12}}{r}-\frac{\sigma^{6}}{r}\right)$$

The *ε *and *σ *values, which are the potential well depth and the distance at which the inter-atomic potential is zero, are experimentally determined and can be retrieved from tables distributed with the Amber-99 forcefield.

### Rigidity theory

**Figure 5:**
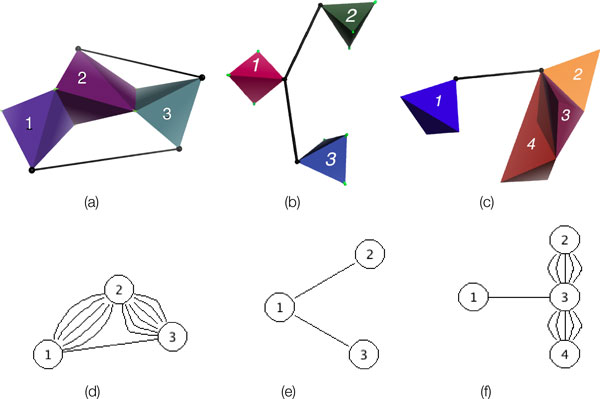
**Examples of generic and non-generic body-bar-hinge frameworks**. Examples of generic and non-generic body-bar-hinge frameworks (a,b,c) and associated graphs (d,e,f). (a) shows a generic body-bar-hinge framework. The bar endpoints, and the continuous sets of points along the hinge axes, are all distinct. Its associated graph, shown in (d), is completely defined. The frameworks of (b,c) contain non-generic features described in this paper: a bar-bar concurrency (b) and a bar-hinge concurrency (c). These two types of degeneracies may occur in mechanical models of proteins when modeling H-bonds or hydrophobic interactions with a bar. Using our heuristic, we build associated graphs for the non-generic frameworks (e,f). Although for these two examples the pebble game will produce the correct result, there is no guarantee for non-generic cases.

A *body-bar-hinge framework *is a 3D mechanical structure made from rigid bodies, pairs of which are connected through hinges and bars. The hinges admit only a rotation of the two incident bodies around the hinge axis. The fixed-length bars connect the bodies at universal joints which allow full rotational freedom. If the only motions of the framework are the trivial rigid motions (those which move the whole system rigidly, maintaining all the pairwise distances between all points), then the framework is said to be rigid. Otherwise it is flexible. The top row of Figure 5 shows a few such examples.

*Combinatorial algorithms for rigidity of body-bar-hinge frameworks*. A rigid body in isolation has 6 trivial degrees of freedom (DOFs): all rigid motions can be generated by translations along the *x, y*, and *z *axes, and by rotations around each of the *x, y*, and *z *axes. Two disconnected rigid bodies have a total of 12 DOFs; *k disconnecte*d rigid bodies have a total of 6*k DOFs*. We associate a multigraph to a body-bar-hinge framework, where each body is represented by a vertex. When two bodies are connected by a bar one DOF is removed. This is represented in the associated multigraph as one edge between the corresponding graph vertices. Adding additional bars between the two bodies can remove up to 6 DOFs, at which point the two rigid bodies are rigidly attached to each other and form a single rigid body. It is not possible to remove the remaining trivial 6 DOFs by placing additional bars or hinge constraints. If two rigid bodies are connected by a hinge joint, 5 DOFs are removed, and 7 DOFs remain. These are the 6 trivial DOFs, and the 1 internal DOF, from the rotation permitted around the hinge axis.

To summarize: the associated graph of a body-bar-hinge framework has 1 vertex for each body, 1 edge for each bar, and 5 edges for each hinge. A simple counting rule, due to Tay [20] (see also [21]) and rigorously proven to be valid by *Tay's theorem*, can then be used on this graph to determine the rigidity and the DOFs of the framework. The (6,6)-pebble game algorithm of [22] runs in *O*(*n*^2^)-time and permits the efficient analysis of this graph, by decomposing it into maximal rigid regions called *rigid clusters*.

*Generic and non-generic mechanical models*. Tay's theorem applies to *almost all *geometric body-and-bar frameworks, but it fails on a statistically insignificant ('measure-zero') set of situations which are called *non-generic *due to the existence of certain algebraic dependencies between the geometric data. Identifying non-generic frameworks is in general a very difficult problem, but it is sometimes possible to state whether certain *combinatorially described *configurations are generic. A famous example is the *Molecular conjecture*, which states that molecular frameworks still obey Tay's theorem, generically, even when the set of hinges incident at an atom are concurrent. This conjecture, essential in establishing the validity of the combinatorial approaches for rigidity analysis of molecular structures, has been proven only recently [23], more than 25 years after it has been raised.

In this paper, we discuss a number of situations, described in combinatorial (rather than geometric) terms and detected from the connectivity of the set of points and constraints, for which a similar *genericity theorem *would be needed. For practical purposes, we will have to work for now under the assumption that the conjecture holds, as this is what allows for the extension of the pebble game algorithm to such cases; otherwise, our implemented method will have to be, for the time being, considered as an unproven heuristic. We point out, however, that such statements are sometimes notoriously difficult to prove; the first result of this kind, due to Maxwell (1864) and Laman (1970) took over 100 years to become a theorem. We will have to resort for now to empirical validation while waiting for the rigorous proofs.

To summarize: 3-dimensions only *generic body-bar-hinge frameworks *can be analyzed using Tay's theorem with theoretical guarantees of correctness. Moreover, in some situations the very definition of the associated graph fails to be well-defined. For example, when the endpoints of two bars coincide, there is no guarantee of them being in a generic position. We call this type of degeneracy a *bar-bar concurrency *(Figure 5b). If one endpoint of a bar lies on a hinge, we have a *bar-hinge concurrency *degeneracy (Figure 5c). How to place the edges in the associated graph for frameworks with this latter type of degeneracy is ambiguous. In the Methods section, we discuss where these degeneracies turn up in protein modeling, and propose a heuristic so that rigidity analysis can be performed.

### Modeling molecules for rigidity analysis

We describe now the modeling core of our software KINARI [3]. It associates to a molecule a body-bar-hinge framework. The bodies, made of rigid groups of atoms, are determined first, then bar and hinge constraints are placed between them. Figure 1 shows how bodies are determined from atoms connected by strong bonds. Strong bonds include covalent bonds, disulfide bonds, and strong H-bonds. Each multi-valent atom, together with its strongly-bonded neighbors, forms a rigid body. The multi-valent atom is the *central atom *for the body, and each body has one unique central atom; the single-valent atoms are *non-central *atoms. When two bodies overlap, as shown in the ethane molecule in Figure 1, the overlap consists of two bonded atoms; they determine an axis which acts as a hinge. When two atoms share a non-rotatable bond, such as a peptide or double covalent bond, an additional bar is placed between the two bodies in the mechanical model to lock the hinge, prohibiting rotation. By default, H-bonds are modeled in the same way as covalent bonds. Hydrophobic interactions are modeled with the heuristic of ASU-FIRST [2], placing an interaction between C-C, C-S, or S-S pairs when when their van der Waals surfaces are within a cutoff distance of 0.25 Å. By default, for each hydrophobic interaction, 2 bars are placed into the mechanical model.

For the remainder of the paper, we refer to our previously released system, with its default options for curation and modeling, as KINARI v1.0 [3]. The KINARI v1.0 results on our data sets will will serve as a baseline in our evaluation.

### Literature review

KINARI is not the only software performing protein rigidity analysis, but there are substantial modeling differences between it and previous ones. We briefly survey their features here.

*Historical perspective*. Protein rigidity analysis, using a pebble game algorithm, was pioneered by Jacobs and Thorpe, and implemented as the MSU-FIRST software [1,24]. The system was upgraded to ASU-FIRST [2], based on a different underlying modeling methodology and on a variation on the pebble game, and was made available on the Flexweb server [25]. The two FIRST versions determined H-bonds using an in-house implementation of geometric criteria and modeled them in the same way as covalent bonds, fixing bond length and angles [1,2]. The software calculated an associated energy for each bond, using an energy function proposed in the Mayo Lab [17], and provided an energy cutoff as a tunable parameter, in order to exclude weaker bonds from the analysis [1]. Later, identification of hydrophobic interactions using a heuristic function was included in the ASU-FIRST software [2].

*Validation of rigidity analysis software*. A few approaches have been taken to validate the biological relevance of the rigidity analysis results. In some of the first studies applying rigidity analysis to proteins, results were qualitatively compared for four proteins: lysine-arginine-ornithine (LAO) binding protein (1lst, 2lao); HIV1-protease (1hhp, 1htg); adenylate kinase (1aky; 1dvr), and dihydrofolate reductase (1ra1, 1rx1, 1rx6). Each of them had multiple structures from unique conformations deposited in the PDB [1,5]. A thorough analysis was provided, comparing their own proposed residue-based flexibility index with PDB B-values, RMSD values, and changes in Φ and Ψ angles. The cluster decomposition itself was analyzed qualitatively, by comparing known flexible domain-level hinge regions with those identified by the software. A later study did not attempt to compare the rigidity results with two conformations, but instead verified the similarity of the rigid cluster decompositions, computed at different H-bond energy cutoffs, of three cytochrome *c *proteins from three different species (1hrc, 1ycc, 1c06 a) [26]. A more quantitative approach was taken to validate dilution, confirming that the folding core identified by dilution agreed with the experimentally determined folding cores of 10 proteins: BPTI, 1bpi; ubiquitin, 1ubi; CI2, 2ci2; ribonuclease T1, 1bu4; cytochrome *c*, 1hrc; barnase, 1a2p; *α*-lactalbumin, 1hml; apo-myoglogin, 1a6m; interleukin-*β*, 1ilb; T4 lysozyme, 3lzm [7]. The StoneHinge method and server for domain-level hinge prediction, developed in the Gerstein Lab, used the MSU-FIRST software as a module [27]. In their study they concluded that the MSU-FIRST-based method over-predicted the occurrence of hinges when compared with a set of literature-annotated hinges. The StoneHinge developers resorted to a consensus-based approach, combining the rigidity analysis results with another independent method, in order to achieve better precision in their predictions. Their data set was composed of: CAPK, 1ctp, 1atp; Bence-Jones protein, 4bjl; LAO-binding protein, 2lao, 1lst; adenylate kinase, 2ak3, 1ake; glutamine binding protein, 1ggg, 1wdn; DNA polymerase *β*, 2bpg, 1bpd; calmodulin, 1cfd, 1cll; inorganic pyrophosphatase, 1k23, 1k20; ribose binding protein, 1urp, 2dri; Ig domain of protein G, 1pdb; hydropterin pyrophosphokinase, 1hka; cyclophilin A, 1bck; rhizopuspepsin, 2apr, 3apr; chloramphenicol acetyltransferase, 2cla, 3cla; and proteinase A, 2sga, 5sga. A more recent study to validate a heuristic version of the pebble game used a decomposition similarity measurement called the Rand Measure [28], which uses counts of the number of items that match and differ between two decompositions, to analyze a data set of 272 proteins with 3 domains or fewer from the SCOP database [29].

*Critical H-bonds and dilution. Althoug*h not included as an option in their software, the makers of MSU-FIRST suggested a H-bond inclusion criterion by determining the set of H-bonds from two conformations of the same protein, and including only those that occurred in both [1] (pg 157, paragraph 3). Kurnikova *et al. propose*d an extension, using snapshots from molecular dynamics simulation data; rather than an energy cutoff, they used the duty cycle, defined as the fraction of time that a particular interaction is present over a number of MD snapshots [30]. Another extension is to perform rigidity analysis at all possible cutoffs, leading to what is called a dilution analysis [7]. It can be interpreted as a simulated unfolding because H-bonds are broken one-by-one, by order of energy. The rigid clusters of the protein are computed at each step, with the most stable part, called the folding core, remaining at the end [7].

*How the present work differs*. To summarize: validation of rigidity analysis as a predictive tool for native state rigid cluster decomposition was previously limited to case studies [1]. Although it was shown that accuracy could be improved by tuning the input set of hydrogen bonds with an energy cutoff, no systematic analysis was performed in order to determine when rigidity analysis worked best and when it fell short. Also, all hydrogen bonds were treated equally and the set of hydrophobic interactions were not varied. In the next section, we introduce our new methods for addressing these limitations.

## Methods

We now describe the three new methods proposed in this paper. We also describe the benchmarking toolkit we wrote for our evaluations and the benchmark data set which we use in our evaluation.

### Modeling weak H-bonds as bars

For our new modeling option for H-bonds, we choose a cutoff energy value, but instead of discarding the weak bonds [1,3], we model them with a weaker constraint than the one used for a covalent bond. In this work, we have chosen to model them mechanically with a bar (described in the Background section), which fixes the distance but permits angles to vary. This bar concept is distinct from the multibar modeling introduced in ASU-FIRST for modeling hydrophobics, where bodies and multiple bars are placed in the model to approximate a pseudo-atom chain [2]. The mechanical models produced may contain degeneracies, as discussed next. Furthermore, we survey where the problems occur for the different types of H-bond configurations we defined in the previous section (Figure 3). For the different H-bond topologies described earlier in the Background section, we describe how they are included in the mechanical modeling and the heuristics needed in order to build a graph for the pebble game algorithm.

*Non-furcated configurations*. Because *H *and *A *are both covalently bonded to only one other atom, during the body-building phase of modeling, they each are placed in one, and only one body. Placing the bar between the two bodies introduces no degeneracies because the endpoints are unique. No other bar is attached at the endpoint.

*Furcated configurations*. When the configuration is a furcated one, either at *H *(Figure 3b) or *A *(Figure 3c), then the mechanical model will contain two bars which share an endpoint. This bar-bar concurrency is combinatorially non-generic, but the multigraph associated to the resulting body-bar-hinge framework is well defined and the usual pebble game can be used in this situation.

*Multiple-base acceptor configurations. A *can be covalently bonded to multiple bases (Figure 3d). In the resulting mechanical model, *A *lies on a hinge and is in more than one body. The mechanical model will contain a bar-hinge concurrency, and the multigraph associated to the resulting body-bar-hinge framework is not uniquely defined.

*Our heuristic*. We propose a heuristic for building the associated graph in the combinatorially non-generic situations identified above. See the Background section for preliminaries. For each bar, if one of its end-points is a non-central atom, then it belongs to only one body and there is no ambiguity: we place an edge in the associated graph that is connected to the vertex corresponding to that body. If a bar's endpoint is attached to a central atom, then, in the multigraph associated to the mechanical structure, we place the edge on the vertex corresponding to the body of the central atom.

### Calculating hydrophobic interactions with energies and modeling as bars

**Figure 6:**
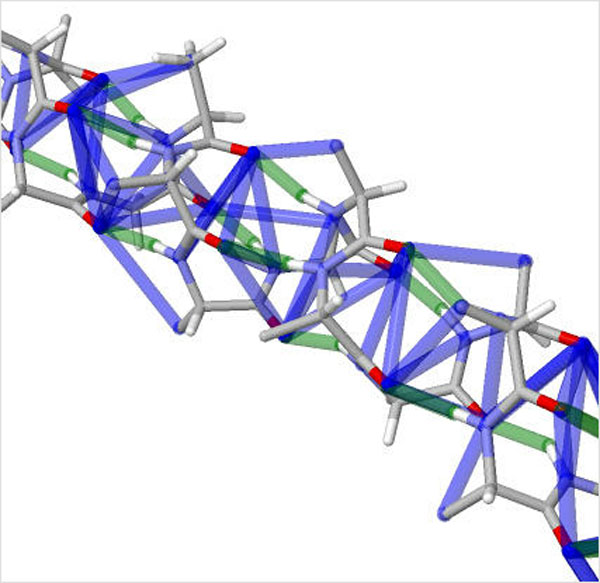
**Calculated noncovalent interactions in an alpha helix**. Hydrogen bonds (green) and hydrophobic interactions (blue) computed on a section of alpha helix. In the 18 residue alpha helix, 14 hydrogen bonds, with energies ranging between -2 and -7 kcal/mol, and 51 hydrophobic interactions, with energies ranging between -0.15 and -0.2 kcal/mol, were identified. With the previous version of calculating hydrophobic interactions in KINARI v1.0, no hydrophobic interactions would be identified within the alpha helix.

Our study is the first to evaluate the effect of varying the hydrophobic interactions on the accuracy of the rigidity results. To provide a tuning parameter for inclusion, we assign an energy to each interaction based on its Lennard-Jones potential, described earlier in the Background section of this paper. The *ε *and *σ *values are taken from the Amber-99 forcefield [19]. Interactions with hydrogen atoms were excluded because these atoms take part in H-bonding. Otherwise, all pairs of atoms, and not just those identified with the heuristic method for hydrophobics as introduced by ASU-FIRST, are considered as candidates for hydrophobic interactions. Figure 6 shows 51 hydrophobic interactions, with energies ranging between -0.15 and -0.2 kcal/mol calculated on an 18-residue alpha helix. The previous version of our software, KINARI v1.0, would determine no hydrophobic interactions in the helix.

To model the hydrophobic interactions, we have chosen the single bar constraint described in the previous section on weak hydrogen bond modeling. This constraint models the atoms' propensity to remain a fixed distance from each other, while permitting angles to vary.

### Comparative cluster decomposition scoring

To evaluate our new methods for including and modeling H-bonds and hydrophobic interactions, we propose the application of a method, borrowed from the information retrieval literature, called B-cubed scoring [4]. The resulting score is a measurement of how well the clusters in the predicted decomposition match some other decomposition determined by some other, different method. For each 'item' (data point, document, residue, etc), the *precision *is the fraction of items in its predicted cluster that also lie in its cluster in the gold standard. The *recall *is the fraction of items in its gold standard which are also in its predicted cluster. The F1-score combines the precision and recall into one score.

*Clustering comparison methods*. Quite a number of methods for comparing clusterings have been previously proposed and compared [31], including Rand Measure [28] and partition-distance [32]. We have chosen B-cubed scoring because it was designed to address shortcomings in prior methods in the treatment of singletons (items which lie in their own unique cluster) [4]. Proper credit for identifying singletons is crucial because in a residue-level rigid cluster decomposition of a protein, flexible regions are composed of such singletons.

*Calculating B-cubed score*. We describe how to compute the B-cubed score. For each item *i, GS*(*i*) is the cluster it belongs to in the gold standard decomposition. Similarly, *M*(*i*) is *i*'s cluster in the model's predicted decomposition. *Pr*(*i*) and *Re*(*i*) are, respectively, *i*'s precision and recall. The precision and recall of a decomposition *D, Pr*(*D*) and *Re*(*D*), are simply the mean precision and recall of the items. *F*1(*D*), the F1-score of *D*, is the harmonic mean of *Pr*(*D*) and *Re*(*D*). The following five equations show how *Pr*(*i*), *Re*(*i*), *Pr*(*D*), *Re*(*D*), and *F *1(*D*) are calculated.

(2) $$Pr\left(i\right)=\frac{|GS\left(i\right)\cap M\left(i)\right|}{\left|M\left(i\right)\right|}$$

(3) $$Re\left(i\right)=\frac{|GS\left(i\right)\cap M\left(i)\right|}{\left|GS\left(i\right)\right|}$$

(4) $$Pr\left(D\right)=\frac{1}{n}\overset{n}{\underset{i=1}{\sum }}Pr\left(i\right)$$

(5) $$Re\left(D\right)=\frac{1}{n}\overset{n}{\underset{i=1}{\sum }}Re\left(i\right)$$

(6) $$F1\left(D\right)=\frac{2*Pr\left(D\right)*Re\left(D\right)}{Pr\left(D\right)+Re\left(D\right)}$$

*All-floppy and all-rigid baselines*. For a set of items, the two most extreme ways of naively decomposing are the all-floppy prediction (placing each item into its own unique cluster) or all-rigid prediction (placing all items into the same cluster). These two methods result in 100% precision and 100% recall, respectively. We will use the all-floppy and all-rigid decompositions as baselines to compare KINARI's decompositions on real proteins.

These baselines might seem rudimentary, but are quite powerful in showing that the higher level of sophistication built into our system provides provably better results. For example, single domain proteins such as dihydrofolate reductase may be mostly rigid, with a small flexible region at the active site. In the KINARI v1.0 decomposition of the open conformation (1ra1), 93% of the residues are contained in the largest rigid cluster. For such cases, the all-rigid baseline will perform better than other methods which err toward a more flexible model.

**Figure 7:**
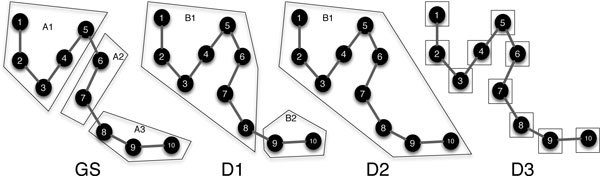
**Example rigid cluster decomposition comparison**. Decompositions on the same example 10-residue protein to demonstrate the cluster decomposition score. GS represents the gold standard decomposition against which predicted decompositions D1-D3 are compared. D2 and D3 are the all-rigid (100%-recall) and all-floppy (100%-precision) decompositions. D1, D2, and D3 receive B-cubed scores, respectively, of 0.65, 0.46, and 0.55. See Table 2 for calculations.

*Example of calculated B-cubed scores*. Figure 7 depicts the predicted decompositions for an abstract molecule, compared with a gold standard decomposition (GS). The decompositions are D1: produced by some predictive method, D2: all-rigid, and D3: all-floppy. The B-cubed scores for D1, D2, and D3, compared with GS, are respectively 0.65, 0.55, and 0.46 (see Table 2 for calculation). This shows that the decomposition of D1, which visually seems to better match the cluster distribution of GS, achieves a higher score than either the all-rigid or all-floppy decomposition.

**Table 2:** Example B-cubed scoring calculations.

	D1		D2		D3	
***Residue***	***Re***	***Pr***	***Re***	***Pr***	***Re***	***Pr***

1	$\frac{5}{5}$	$\frac{5}{8}$	$\frac{5}{5}$	$\frac{5}{10}$	$\frac{1}{5}$	$\frac{1}{1}$

2	$\frac{5}{5}$	$\frac{5}{8}$	$\frac{5}{5}$	$\frac{5}{10}$	$\frac{1}{5}$	$\frac{1}{1}$

3	$\frac{5}{8}$	$\frac{5}{5}$	$\frac{5}{10}$	$\frac{1}{5}$	$\frac{1}{1}$	$\frac{5}{5}$

4	$\frac{5}{5}$	$\frac{5}{8}$	$\frac{5}{5}$	$\frac{5}{10}$	$\frac{1}{5}$	$\frac{1}{1}$

5	$\frac{5}{5}$	$\frac{5}{8}$	$\frac{5}{5}$	$\frac{5}{10}$	$\frac{1}{5}$	$\frac{1}{1}$

6	$\frac{2}{2}$	$\frac{2}{8}$	$\frac{2}{2}$	$\frac{2}{10}$	$\frac{1}{2}$	$\frac{1}{1}$

7	$\frac{2}{2}$	$\frac{2}{8}$	$\frac{2}{2}$	$\frac{2}{10}$	$\frac{1}{2}$	$\frac{1}{1}$

8	$\frac{1}{3}$	$\frac{1}{8}$	$\frac{3}{3}$	$\frac{3}{10}$	$\frac{1}{3}$	$\frac{1}{1}$

9	$\frac{2}{2}$	$\frac{2}{3}$	$\frac{3}{3}$	$\frac{3}{10}$	$\frac{1}{3}$	$\frac{1}{1}$

10	$\frac{2}{2}$	$\frac{2}{3}$	$\frac{3}{3}$	$\frac{3}{10}$	$\frac{1}{3}$	$\frac{1}{1}$

Avg	0.93	0.51	1.0	0.38	0.30	1.0

F1	0.65		0.55		0.46	

Shown are the calculations of B-cubed precision, recall, and F1-scores for the small examples shown in Figure 7. The scoring method is described in the Methods section. The B-cubed score (shown as F1) for decomposition D1 is higher than the all-rigid (D2) and all-floppy (D3) decompositions. In the all-rigid baseline, all 10 residues are placed into the same cluster (D2), resulting in 100% recall but low precision. To contrast, in the all-floppy baseline, each residue is placed in a unique cluster resulting in 100% precision but low recall.

### Benchmarking toolkit

We have developed scripts for benchmarking protein decompositions sytems (not just those that rely on pebble game rigidity analysis). The main scripts are:

scoreRigidityResults.py : takes as input gold standard and predicted decompositions and outputs the B-cubed precision, recall, and F1-score

getNaiveBCubedScores.py : takes as input a gold standard decomposition and outputs the B-cubed precision, recall, and F1-score for the all-rigid and all-floppy decompositions to use as baselines.

The ingredients for using the benchmarking toolkit are, first, a data set of PDBs with some associated gold standard cluster decompositions and, second, predicted decompositions on the same data set of proteins. As input, the scripts support the file format produced by RigidFinder for defining the decompositions into sets of residues. The scripts, data sets, and baselines are available at the KINARI website Downloads page [3].

### Benchmark data set

**Figure 8:**
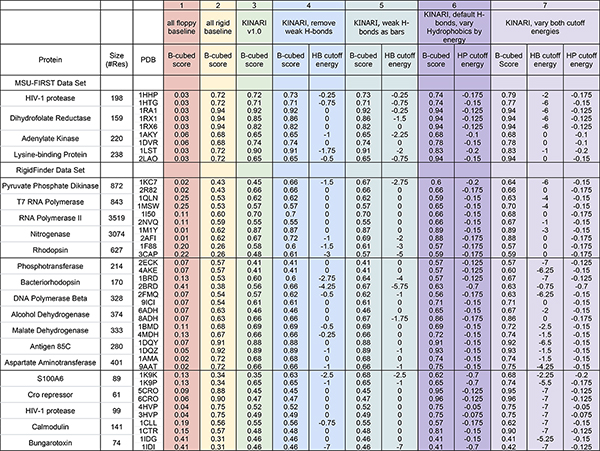
**Results of B-cubed scoring evaluation on benchmark data set**. We determined rigid cluster decompositions for each PDB file in the benchmark data set, using the 7 methods listed in Table 3. The data set comes from publications by two different software for performing rigid cluster decompositions. The MSU-FIRST portion of the data set consists of 4 proteins used to validate the MSU-FIRST software [1,5]. The RigidFinder portion of the data set is categorized, from top to bottom, into large (greater than 500 residues), medium (between 200 and 500 residues) and small (fewer than 200 residues) proteins. Decomposition methods 1 through 7 are summarized in Table 3. For each method, the maximum B-cubed score and corresponding hydrogen bond and hydrophobic energy cutoffs (where relevant) are shown. See Figure 9 for barplots comparing each method against methods 2 and 3.

*Gerstein Lab RigidFinder data set*. To apply B-cubed scoring to real proteins, 'gold standard' reference decompositions are needed. The Gerstein Lab's data set, listed in Figure 8, was used to validate the RigidFinder method, which determines rigid cluster decompositions using two conformations of the same protein [6]. The decompositions for these are readily available from the RigidFinder website, and have been well tested and validated against evidence from the biochemistry literature. The data set has good coverage over small (fewer than 200 residues), medium (between 200 and 500 residues), and large (greater than 500 residues) proteins. Due to limitations in the PDB format, we have excluded GroEL-GroES from our study. The RigidFinder-computed decompositions for the 16 proteins are used as the gold standard to compare our results using KINARI.

*MSU-FIRST data set*. In order to compare the new modeling options against previous results, we include four proteins used in the validation of the MSU-FIRST software: the LAO binding protein (closed, 1lst; open, 2lao), HIV-1 protease (closed, 1hhp; open, 3phv), dihydrofolate reductase (open, 1ra1; closed, 1rx1; occluded, 1rx1), and adenylate kinase (open, 1dvr; closed, 1aky) [1,5]. There is an overlap between these proteins and those in the RigidFinder data set because these are standard, well-studied proteins for which multiple conformations are known. We used RigidFinder to determine our gold standard rigid cluster decompositions, choosing the decomposition according to the established convention, choosing the first sensitivity cutoff at a local maximum [6].

*Converting to residue-level clusters*. KINARI employs an all-atom model to determine rigid clusters of atoms. Because the decompositions for the Gerstein Lab's benchmark data are instead at the residue level, the KINARI output must be transformed. To do this, we examine the body-bar-hinge model output by KINARI. For each atom-level rigid cluster of determined by KINARI, we first create an empty residue-level cluster and then collect the residues whose CA atoms belong to the cluster. Note that because rigid clusters can overlap, the CA atoms do not necessarily belong uniquely to that cluster. For each such CA atom, we examine the C-CA and CA-N bonds. If neither corresponds to a hinge in the body-bar-hinge model, meaning that the rotation is inhibited by the network of chemical constraints, we add the CA atom's residue to the residue cluster. Finally, the residue-level cluster is added to the decomposition to be compared with the benchmark.

## Results and discussion

First, in order to validate whether the results match those previously published for MSU-FIRST, we have provided case study analyses to compare the results of KINARI v1.0 with the four proteins published in the MSU-FIRST studies [1,5].

**Figure 9:**
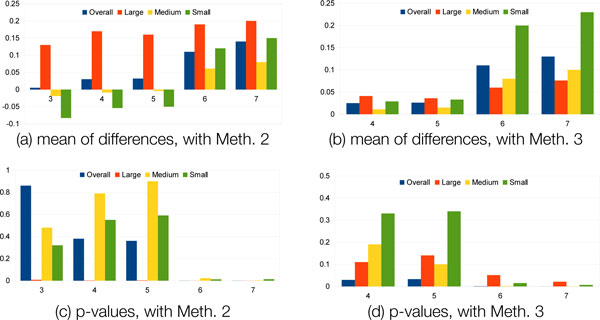
**Baseline comparison of B-cubed scores of RigidFinder data set**. Mean of differences and p-values for each decomposition method (listed in Table 3), compared with all-rigid baseline (method 2) and KINARI v1.0 (method 3). Results include only the RigidFinder portion of the data set (see Figure 8). The mean of differences measures the change in B-cubed score between the two methods; a better-performing method will have a higher associated mean of differences. The p-value indicates whether the improvement is significant (p-value≤ 0.05 is deemed significant). The greatest improvement in B-cubed scores, most significantly in the small to medium sized proteins, resulted when both the modeling of hydrogen bonds and hydrophobic interactions were varied (method 7).

Then, in the sections that follow, we will discuss the results applying the B-cubed scoring benchmarking methodology to methods 3 through 7, summarized in Table 3. All scores and their associated cutoffs are listed in Figure 8. The bar-plots in Figure 9 show the comparisons between each of the methods with method 2 (completely rigid decomposition) and method 3 (KINARI v1.0). The plots show the means of differences and the p-values. We have also included two in-depth case studies on pyruvate phosphate dikinase and calmodulin, in order to demonstrate the sensitivity of the B-cubed evaluation method.

**Table 3:** Decomposition methods.

Decomposition Method	Description
1	All-floppy decomposition

2	All-rigid decomposition

3	KINARI v1.0, default options

4	KINARI, vary hydrogen bond energy cutoff and exclude weak hydrogen bonds

5	KINARI, vary hydrogen bond energy cutoff and model weak hydrogen bonds as bars

6	KINARI, use default options for hydrogen bonds. compute hydrophobics and assign energy with LJ-potential. Exclude weak hphobes and model the rest as bars

7	KINARI, same as Method 6, but vary the hydrogen bond energy cutoff and model the weaker hydrogen bonds as bars

We evaluate the 6 decomposition method variants. The first three serve as baselines against which the new modeling options, proposed in this paper, are compared. The data set and results of of our evaluation of the 7 methods are listed in Figure 8.

### Evaluation of KINARI v1.0 in case studies

We describe the results of KINARI v1.0, with default options, compared with those reported in the MSU-FIRST software on the 4 proteins [1,5]. We found that overall, the cluster decompositions produced by the two methods, visually, had high overlap in the rigid clusters and flexible regions identified. The MSU-FIRST report confirms literature annotated flexible loops with those identified by the software. For most of the cases, KINARI identifies the same flexible loops as does MSU-FIRST. Table 4 summarizes the counts of loops detected by MSU-FIRST and matched by KINARI.

**Table 4:** Comparison of flexible loops detected by MSU-FIRST, KINARI v1.0, and RigidFinder on four proteins.

Protein	PDB	MSU-FIRST	KINARI v1.0	RigidFinder
LAO-binding 1 LAO-binding 2	1lst 2lao	1 1	1 1	1 1

HIV-1 Protease 1 HIV-1 Protease 2	1hhp 1htg	3 4	3 3	2 2

Dihydrofolate Reductase 1 Dihydrofolate Reductase 2 Dihydrofolate Reductase 3	1ra1 1rx1 1rx6	2 2 2	2 1 2	2 2 2

Adenylate Kinase 1 Adenylate Kinase 2	1aky 1dvr	6 4	6 4	3 3

A comparison of the flexible loop regions detected by KINARI v1.0, MSU-FIRST, and RigidFinder. The four proteins, with annotated flexible loops, were used in the validation of MSU-FIRST [1,5]. The table data for MSU-FIRST was taken from the same publications. For KINARI v1.0 and RigidFinder, flexible loops were determined visually. Because RigidFinder computes decompositions based on multiple conformations, the results shown will match for all conformations of the same protein.

Next, we provide in-depth case studies on the four proteins in the MSU-FIRST study [1,5].

#### Case study of Lysine-Arginine-Ornithine Binding Protein

**Figure 10:**
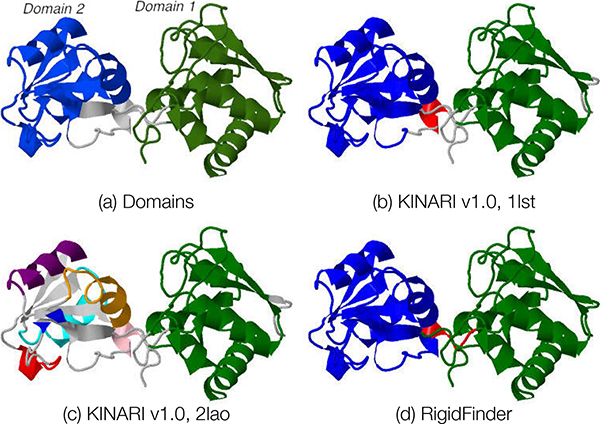
**Rigid cluster decomposition of lysine-arginine-ornithine binding protein**. All of the decompositions are depicted on 2lao. (a) LAO-binding protein is composed of two functional domains. (b,c) Decompositions for 1lst and 2lao computed via KINARI v1.0. (c) Decomposition produced by RigidFinder computing using both conformations.

The lysine-arginine-ornithine binding protein (LAO), which transports important substrates in bacteria, has a bi-lobal or 'clam-shell', structure. The two LAO crystal structures used in the original MSU-FIRST study were an open (2lao) and closed (1lst) structures [5]. It is composed of two stable domains shown in Figure 10a: domain 1 (residues 1-87, 195-237, containing N- and C- terminals) and domain 2 (residues 94-181). The remaining region consists of loops forming a domain-level hinge.

The MSU-FIRST results report that the residues of domain 1 lie in a single rigid cluster. It was observed that domain 2 is more flexible. Smaller rigid cluster, mainly composed of *α*-helices, form within the domain, but the *β*-sheet remains flexible. There are slight differences in the distribution of the rigid clusters between the open and closed conformations, but for both conformations, the MSU-FIRST software predicts domain 2 to be rather flexible. The main difference between the two decompositions was that for the open conformation, the flexible domain-level hinge region is larger, extending further into domain 2 than for the closed conformation. The open conformation is expected to be more flexible than the closed conformation because the interfaces are separated and there are fewer opportunities for hydrogen bonding and hydrophobic interactions.

In the KINARI decompositions, the difference in flexibility between the open and closed conformations is more stark. The KINARI decomposition on the closed conformation (2lao, Figure 10c) reflects the same level of flexibility as that produced by MSU-FIRST. Like the MSU-FIRST decomposition, domain 1 lies in a single rigid cluster while domain 2 is composed of smaller, mainly *α*-helix, rigid clusters and a flexible *β*-sheet. The decomposition for the closed conformation (1lst, Figure 10b) does not show the same flexibility in the 2nd domain. The two domains are both placed into their own rigid clusters. Thuse, the variations within the two systems results in some subtle differences for the cluster decompositions between the open and closed structures.

For another comparison, we used RigidFinder [6] to decompose the protein into rigid domains, based on the open and closed conformations. RigidFinder decomposes the protein into exactly three domains: one that matches domain 1, another that matches domain 2, and a third composed of the domain-level hinge region (Figure d). The RigidFinder decomposition does not identify the flexibility within domain 2.

##### Case study of HIV-1 Protease

**Figure 11:**
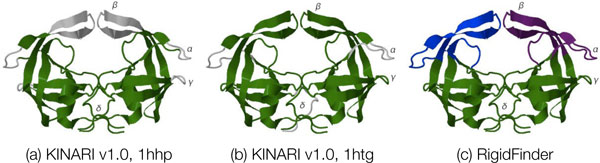
**Rigid cluster decompositions of HIV-1 Protease**. All of the decompositions are depicted on the 1hhp dimer. (a,b) Decompositions for 1hhp and 1htg computed via KINARI v1.0. (c) Decomposition produced by RigidFinder computing using both conformations.

For the open and closed forms of HIV-1 Protease (1hhp, 1htg), the results reported by MSU-FIRST and KINARI have good correspondence. The KINARI v1.0 decompositions for the two conformations are shown in Figure 11a-b. For the closed conformation, the KINARI residue-level cluster decomposition was the same whether the ligand was present or removed from the analysis. For 1hhp, both MSU-FIRST and KINARI identify the single, dominating rigid cluster and the three flexible regions (labeled as *α, β*, and *γ*), Figure 11a. The large rigid cluster contains the base and walls of the binding cavity. For the closed form (1htg), KINARI and MSU-FIRST results both reflect the increase in rigidity upon binding. The large rigid cluster now includes the *α *and *β *regions, but not the *γ *region.

One interesting difference is in the *δ regio*n (the dimer interface) composed of residues at the N- and C-termini that do not belong to a secondary structure. Although the KINARI results show flexibility in the *δ region *in chain A, chain B has maintained rigidity. In the MSU-FIRST decomposition, the entire *δ regio*n is flexible. The loops above the *δ region *is the catalytic site, containing the characteristic Asp-Thr-Gly sequence (Asp25, Thr26 and Gly27) common to aspartic proteases, lie within the rigid core for both the open and closed conformations (computed both by KINARI v1.0 and ASU-FIRST). Functionally, the *δ regio*n is where the two monomers are held together; none of the many known drug resistance mutation sites are located within *δ *[33]. The importance of the findings on the different levels of flexibility within the regions is unclear.

The RigidFinder decomposition for HIV-1 Protease (taken from the RigidFinder data set described in the Methods section of this paper), depicted in Figure 11c, has some interesting differences from the either of the KINARI or MSU-FIRST decompositions. The *α *and *γ *regions, the *β*-sheet that both regions share, as well as loops forming the wall of the binding cavity, have been placed into a single cluster. The rest of the protein, including *γ *and *δ *regions compose a second rigid cluster.

##### Case study of Dihydrofolate Reductase

We compare the MSU-FIRST decompositions of 3 conformational states of dihydrofolate reductase (open, 1ra1; closed, 1rx1; occluded, 1rx5) with those from KINARI v1.0. The KINARI v1.0 decompositions, depicted in Figure 11a-c, were overall much more rigid than those reported by MSU-FIRST. The labeled M20 and *β*F-*β*G loops are of key importance to binding specificity and should be flexible. Overall, the KINARI results report a larger dominating rigid cluster. Even with the exclusion of the ligand from the analysis, the entire protein, other than the 2-3 flexible loops reported, is included in a single rigid cluster. We now compare in further detail the results reported by MSU-FIRST and KINARI in detecting flexibility in the loop regions.

For 1ra1, MSU-FIRST correctly detects flexibility in the M20 loop and captures the flexibility of a subsection of the *β*F-*β*G loop. KINARI detects the same regions of flexibility as MSU-FIRST on 1ra1.

For 1rx1, part of the M20 loop is detected by MSU-FIRST to be flexible, while most of the *β*F-*β*G loop is detected to be flexible. KINARI does not detect any flexibility in the M20 loop, but detects the same flexible region as MSU-FIRST in the *β*F-*β*G loop.

The MSU-FIRST results for 1rx6 are similar to those it reports for 1rx1, but an even larger region of flexibility is detected in the *β*F-*β*G loop. The flexible region of the M20 loop is more extensive (labeled *δ*) and the entire *β*F-*β*G loop is detected to be flexible.

**Figure 12:**
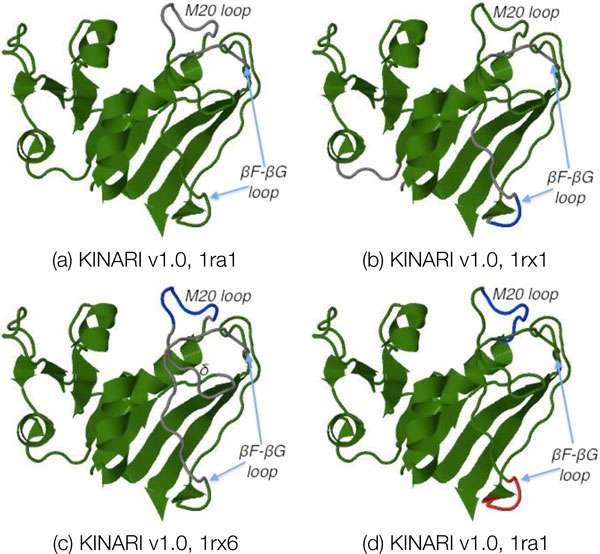
**Rigid cluster decompositions of Dihydrofolate Reductase**. All of the decompositions are depicted on 1ra1. (a,b,c) Decompositions for 1ra1, 1rx1, and 1rx6 computed via KINARI v1.0. (d) Decomposition produced by RigidFinder computing using 1ra1 and 1rx6 conformations.

The RigidFinder decomposition, based on 1ra1 and 1rx1 and shown in Figure 12d, places most of the protein into a single domain, except for a piece of the M20 loop and a small segment of loop adjacent to the *β*F-*β*G loop.

##### Case study of Adenylate Kinase

**Figure 13:**
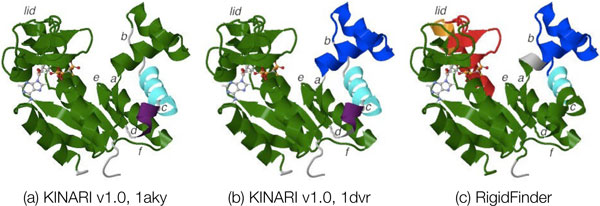
**Rigid cluster decompositions of Adenylate Kinase**. All of the decompositions are depicted on 1dvr. (a,b) Decompositions for 1aky and 1dvr computed via KINARI v1.0. (c) Decomposition produced by RigidFinder computing using both conformations.

Adenylate kinase undergoes a domain-level hinge motion upon ligand binding. Figure 13 shows decompositions of adenylate kinase, depicted on the ATP-bound, open conformation (1dvr). The domain containing the binding domain is labeled as the lid-domain. The *AP*_5_*A*-bound, fully-closed conformation (1aky, not shown) was analyzed.

For 1aky, MSU-FIRST and KINARI v1.0 decompositions match quite closely, with a nice alignment between the clusters and flexible regions between the two decompositions. However for the open conformation (1dvr), there are some subtle differences between the two decompositions. MSU-FIRST identifies 6 flexible loop regions (labeled a-e). Of these, KINARI's flexible regions match with all but 2 of them. Instead, KINARI includes these two loop regions (labeled as e and f) in rigid clusters. KINARI identifies the entire lid region as rigid, while in MSU-FIRST, the loops on the tip of the region have been identified as flexible. The KINARI v1.0 decompositions are shown in Figure 13a-b.

Figure 13c shows the RigidFinder decomposition, determined using both conformations (1aky, 1dvr). RigidFinder detects more flexibility in the LID region than KINARI v1.0. The method captures flexibility at the labeled loops a-c, but he loop regions d-f are contained in the largest rigid cluster.

#### Cluster decomposition evaluation with decomposition method 3, KINARI v1.0

We applied the B-cubed scoring method to the decompositions produced by KINARI v1.0 on the benchmark dataset.

Because the RigidFinder decompositions for the 4 proteins studied by MSU-FIRST were not readily availab, we used the RigidFinder server to generate a residue-level cluster decomposition for each of the four proteins in the MSU-FIRST study [1,5], using the two conformations for each protein as input. Figure 8 shows the results of our evaluation using the B-cubed evaluation method. For 3 of the proteins, KINARI v1.0's score matched or performed better than the all-rigid baseline, for either the open or closed conformations. The RigidFinder decomposition for dihydrofolate reductase is quite rigid, with over 90% of residues lying in the largest rigid cluster. Although KINARI v1.0 detected the flexible loops for 1ra1 (see Figure 12), the RigidFinder decomposition detected only one of them. A different tuning value for the RigidFinder method, with a more flexible decomposition, may result in improved B-cubed scores for KINARI v1.0.

Next, we used KINARI v1.0 to compute a rigid cluster decomposition for the 32 PDBs in the Gerstein Lab data set. For 11 of the 17 proteins, the B-cubed score for at least one of the conformations was higher than that of the completely rigid baseline. For some of the proteins, there was a large discrepancy between the B-cubed scores. For example, the closed (1kc7) and open (2r82) conformations of pyruvate phosphate dikinase received scores of 0.45 and 0.66, respectively.

We performed a paired t-test on the results from the RigidFinder data set in order to evaluate whether improvement was significant over a crude baseline (method 2). The means of differences and p-values are shown in Figure 9. This was indeed the case for the large proteins in the set (p-value, 0.0077), but overall, the improvement over the baseline was not statistically significant. For the PDBs of medium and small proteins, the mean of the differences in B-cubed was negative, showing that a completely rigid decomposition was a better method prediction of the true decomposition. For example, the KINARI v1.0 decomposition on antigen 85C (1dqz) received a B-cubed score of 0.89, the highest among the Gerstein Lab data set. But method 2 received an even higher score, 0.92.

In summary, KINARI v1.0 can produce significant results for large proteins, but for medium and small proteins in the data set, the results are not significant. In the next sections, we explore different parameterizations of the rigidity analysis and how these may improve accuracy.

#### Cluster decomposition evaluation with decomposition method 4, discarding weak H-bonds

For each of the PDBs, we compute the cluster decomposition score for the rigidity results produced at each H-bond energy cutoff, excluding weaker H-bonds. This is the conventional tuning parameter used in previous studies using rigidity analysis [11]. The highest score was determined with its associated cutoff. If multiple cutoffs achieved the same score, the strongest (most negative) cutoff was the one used. The values are listed in Figure 8.

For 11 of the PDBs (1hhp, 1rx1, 1lst, 1kc7, 2afi, 1f88, 3cap, 2brd, 2fmq, 1k9k, 1cll), excluding weaker H-bonds resulted in higher B-cubed scores than KINARI v1.0, in a few cases, quite substantially. This seemed to be the case when there was a large discrepancy in the KINARI v1.0 between two conformations of the same protein, as is typical for open and closed conformations. Removing H-bonds from the more rigid conformation results in a decomposition that more closely matches those of RigidFinder and the other conformation in the pair. The case study of pyruvate phosphate dikinase described in the next section will illustrate this situation.

#### Cluster decomposition evaluation with decomposition method 5, modeling weak H-bonds as bars

We reran our rigidity analysis experiments with KINARI, with the new proposed modeling method for weak H-bonds described in the Methods section. For those PDBs for which using a cutoff did not lead to a higher score, the results were the same. For the 12 which benefited from the cutoff, 5 PDBs received higher scores, three did worse, and the rest remained unchanged.

The MSU-FIRST and Gerstein Lab's benchmark data sets of 21 proteins are insufficient for inferring general conclusions on whether the new modeling H-bond method is significantly better than the default modeling method (removing weak H-bonds). One of the tasks that should be undertaken in the future is to collect and validate a larger benchmarking data set; this task is beyond the scope of this paper.

##### Case study of pyruvate phosphate dikinase

**Figure 14:**
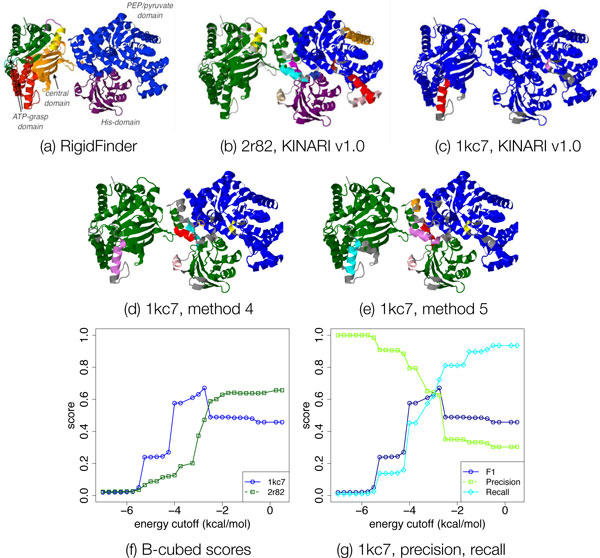
**Case Study of Pyruvate Phosphate Dikinase**. In this case study, we demonstrate how B-cubed scoring may be used to determine the parameter settings for rigidity analysis. (a) shows the RigidFinder decompositions of PPDK, which was validated against literature-annotated functional domains [6]. The KINARI v1.0 decompositions of the open (2r82) and closed (1kc7), shown in (b) and (c), have B-cubed scores of 0.66 and 0.45 respectively. By varying the H-bond energy cutoff (method 2 in Table 3), decompositions with higher B-cubed scores for 1kc7 could be generated. The KINARI decomposition for 2r82 was optimal at cutoff energy 0, meaning all H-bonds were included. For 1kc7, the maximum score, 0.66, was attained at -1.5 kcal/mol when excluding weak H-bonds from the modeling (d). By using a bar to model weak H-bonds, a slightly better score (0.67, cutoff -2.75 kcal/mol) was achieved (e). The B-cubed score plots for the two conformations, using method 7, are shown in (f). As the cutoff is varied, the precision and recall are monotonically increasing and decreasing, shown in (g). An optimal B-cubed score is achieved when the F1-values combining the precision and recall is optimized.

Pyruvate phosphate dikinase (PPDK) is a catalytic-enzyme which binds with ATP, pyruvate, and phosphate. The cluster decomposition produced by RigidFinder and KINARI v1.0 on the open (2r82) and closed (1kc7) conformations, are shown in Figure 14a-c. Visually, the 2r82 decomposition (Figure 14b) shows better agreement with that of RigidFinder's. A segmentation of the PEP/Pyruvate and His domains has been correctly identified. The ATP-grasp domain does not appear in its own cluster. The decomposition for the closed conformation (Figure 14c, 3D depiction of conformation not shown) placed most of the protein into the same rigid cluster. A small fragment, a single alpha helix, of the ATP-grasp domain, has been determined to lie in a different rigid cluster. The all-floppy and all-rigid decompositions have scores of 0.02 and 0.43. Both the KINARI decompositions attained better scores: 0.65 for 2r82 and 0.45 for 1kc7. The difference in scores between the two KINARI decompositions reflects the better accuracy of the decomposition for 2r82. The more rigid prediction for 1kc7 is not surprising, given that it had 10% more H-bonds and 14% more hydrophobic interactions than 2r82.

By excluding weaker H-bonds from 1kc7, as in method 4, a decomposition which more closely matches the RigidFinder decomposition is attained. Figure 14f shows the F1-scores for the decompositions produced by method 4 for each H-bond energy cutoff. For 2r82, the match is optimal at a cutoff of 0 kcal/mol, where all H-bonds are retained. For 1kc7, excluding H-bonds weaker than -1.5 kcal/mol results in the optimal score of 0.66. The corresponding decomposition is shown in Figure 14d, which places the important functional domains into separate rigid clusters. Applying method 5 (bar modeling) to 1kc7 results in a higher B-cubed score of 0.67 at the optimal cutoff of -2.75 kcal/mol, see Figure 14e.

This example on PPDK shows that by calculating the B-cubed score, the optimal cutoff can be determined automatically. Although there is no universal parameterization for rigidity analysis, future studies should explore under what conditions, such as conformational state or active temperature, the same cutoff best applies.

*Prevalence of degeneracies in mechanical models*. Because they are usually less linear, H-bonds in furcated configurations tend to have weaker energies. They are more likely to be left-out from the mechanical model if an energy cutoff is used. Furcated bonds are bundled together (by definition), so removing them can have a drastic impact on the rigidity of a local area. By modeling them as a bar, we can more realistically capture them in the model as weaker then covalent bonds. Although individually the bars make a smaller contribution, when taken together, they have a significant effect on the rigidity.

Furcated configurations do introduce bar-bar concurrency degeneracies into the model, and depending on the boundary chosen between weak and strong, bar-bar degeneracies may be in abundance in the mechanical model. There is a sterically-imposed bound on the number of H-bonds in a furcated configuration. Although [14] found examples of up to hexafurcated configurations, these were rare, and most configurations were bifurcated or trifurcated.

All bar-hinge concurrencies in the mechanical model are introduced when modeling H-bonds in multi-base acceptor configurations. These H-bonds tend to be less common (about 6% of the H-bonds in our analysis, see Figure 4 and Table 1) and when they do occur, they are stronger (Figure 4).

#### Cluster decomposition evaluation with decomposition method 6, when using hydrophobic interaction energy cutoff

We repeated the evaluation, but this time, we used our new methods for hydrophobic interaction identification and modeling, as described in the Methods section. All identified H-bonds were included and modeled with the default modeling option, but the hydrophobic interaction energy cutoff was varied.

For the RigidFinder data set, the improvement in the B-cubed scores over the baselines, methods 2 and 3, was significant. Compared with method 3, the mean of differences over the set of PDBs was 0.11 overall, and the p-value in the paired t-test was 0.00071 (Figure 9). The improvement was near-significant for the large proteins (p-value 0.051), and significant for the medium and small-sized proteins (p-values 0.0015 and 0.015). For the majority of proteins, the change in the hydrophobic modeling improved B-cubed scores. There was no consensus in the best energy cutoff value, but the median was -0.15 kcal/mol.

#### Cluster decomposition evaluation with decomposition method 7, varying both hydrogen bond energy and hydrophobic interaction energy cutoff

We next varied the energy cutoffs for both H-bonds and hydrophobic interactions. Hydrophobics were included and modeled with the same scheme as in the method 6. For H-bonds, we modeled bonds weaker than the cutoff with a 'bar' constraint (rather than excluding), as in method 5.

Compared with the highest scores attained on each PDB over all previous methods, method 7 achieved an improvement in over 70% of the 43 PDB files. The average change in score over method 6 was 0.02, and for a few cases, such as S100A6 and calmodulin, the increase in score was quite significant. As with the previous method, the median cutoff for hydrophobic interactions was again -0.15 kcal/mol. For the medium and large proteins in the data set, including some or all H-bonds achieved the best score, confirming what has been stated in the literature [10] that the best results come from a balance between the two types of stabilizing interaction. For 4 out of 5 of the small proteins, including no H-bonds (shown with a cutoff energy of -7 kcal/mol), but still excluding some hydrophobic interactions, produced the best decompositions.

We have introduced a method for identifying hydrophobic interactions and assigning energies, using the Lennard-Jones potential. This is the first study to formally evaluate how the set of hydrophobics included can impact the rigidity results. Although it has been mentioned in papers from the Gohlke lab [10,34] that the hydrophobic identification function was insufficient for achieving valid rigidity results for some classes of molecules (for example, RNA), there has been no thorough study in order to determine the best parameterization for hydrophobics. Ours is the first study to try to improve upon the inclusion of hydrophobic interactions.

**Figure 15:**
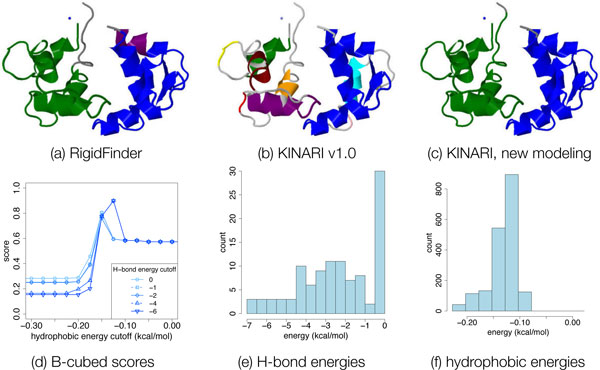
**Case study of Calmodulin (1ctr)**. In this case study, we demonstrate how varying the H-bonds and hydrophobic interactions included can produced a cluster decomposition that better matches with the 'gold standard'. (a-c) Rigid cluster decompositions of 1ctr by RigidFinder, KINARI v1.0 (method 3), and by varying hydrogen bonds and hydrophobic interactions (method 7). Using the RigidFinder decomposition as the gold standard for comparison, the decompositions of (b) and (c) attained, respectively, B-cubed scores of 0.48 and 0.90. (d) shows a plot of the B-cubed score as the energy cutoffs for H-bonds and hydrophobic interactions are varied, via method 7. To show how the H-bond and hydrophobic interaction energies are distributed, we include figures (e) and (f). The KINARI-Web server provides the functionality of viewing such histograms when choosing energy cutoffs.

*Case study of calmodulin*. Figure 15d shows the results of varying both hydrogen bond energy and hydrophobic energy cutoff for calmodulin (1ctr). By removing H-bonds and adding hydrophobic interactions (energy histograms shown in Figure 15e,f), an improved fit with the gold standard decomposition (shown in 15a) is achieved. Run with KINARI v1.0 (using its default options, Figure 15b), the B-cubed score of 0.48 was worse than the score of the all-rigid baseline. By removing some or all H-bonds and including some hydrophobic interactions (decomposition in Figure 15c), a score of 0.90 is attained.

#### Further discussion

*Gold standard data sets*. A major challenge in evaluating the performance of a rigidity analysis system is in finding a high quality data set, grounded in laboratory experimental results, to serve as the gold standard. Within the RigidFinder data set which we used as our gold standard decompositions, we found that there is some disagreement in the rigid and flexible domains as compared with those annotated by the authors of MSU-FIRST [1]. RigidFinder is very effective at determining course-grained domain decompositions, but lacks the sensitivity to identify smaller flexible loops that may be functionally very important. This issue can be observed in decompositions of adenylate kinase (1aky, 1dvr), which has 6 functionally important flexible loop regions as annotated in [1] (see also Table 4 and Figure 13). RigidFinder places the loops into their adjacent rigid clusters.

*Atom-level decomposition*. The evaluations were performed only on backbone flexibility, not taking advantage of the atom-level decompositions produced by KINARI. If such a benchmarking data set were available, it is within the power of our evaluation framework to compare sidechain flexibility as well.

*H-bond classification*. Our H-bond identification and classification method, using HBPLUS and the Mayo energy function, has its limitations. It would also be interesting to use different criteria to classify the bonds as weak and strong, for example, the duty cycle of Kurnikova *et al*. [30] or our classification of H-bonds as critical and redundant [35].

*Evaluating the heuristic approach to handle degeneracies in the model*. We have proposed a heuristic for placing the edges into the associated graph for non-generic bars in the mechanical model. To analyze, empirically and mathematically, when the heuristic works and when it fails is a problem for future investigations.

## Conclusions

As has been iterated through the literature and demonstrated in this paper, a one-size-fits-all parameterization for rigidity analysis does not deliver good across-the-board performance. Some tuning may be required to attain a rigid cluster decomposition for a protein that most closely agrees with data from experimental studies. In this paper, we proposed three new methods: one for for inclusion and modeling of hydrogen bonds, a second for the inclusion and modeling of hydrophobic interactions, and third, a cluster decomposition evaluation method. We showed on a benchmarking data set that the new modeling in KINARI can produce rigid cluster decompositions, computed on single conformation, that better match gold standard decompositions than previous methods. To do this, we applied a comparative decomposition scoring algorithm, first used in information retrieval, called the B-cubed score.

The power of a benchmark lies in fast hypothesis testing, which we demonstrated first by proposing new modeling methodologies for hydrophobic interactions and weak hydrogen bonds. The greatest improvement in B-cubed scores, most significantly in the small to medium sized proteins, resulted when both the modeling of hydrogen bonds and hydrophobic interactions were varied. This supports an earlier insight made by Gohlke *et al*. that "Finding the appropriate balance between these interactions [H-bonds and hydrophobics] is thus crucial for an accurate representation of the flexibility characteristics of proteins".

Because we had access to decompositions that were well-validated against experimental studies, we were able to find combinations of parameters which maximized the B-cubed score and therefore the matching with the gold standard. Computing the B-cubed score at multiple cutoffs helps in choosing the best cutoff, so the score can be used to automatically tune parameters using a training set. To improve pebble game rigidity analysis as a predictive tool, the next steps should be toward finding the relevant features of a protein and its PDB structure which will lead to the best tuning of the parameters to capture biologically important characteristics in the decomposition. At a minimum, the features should include the size of the protein and whether the conformation is open or closed.

## List of abbreviations used

DOFs is used for "degrees of freedom". H-bonds is used for "hydrogen bonds". *A *and *D *are used for the "acceptor" and "donor" atoms in a hydrogen bond. GS is used for "gold standard".

## Competing interests

The authors declare that they have no competing interests.

## Authors' contributions

NF designed and ran the experiments and wrote the supporting code. IS oversaw all work and designed the modeling scheme of the KINARI software. Both authors drafted the manuscript, and read and approved the final version.

This article is an expanded version of an extended abstract that appeared previously, which focused on the modeling of hydrogen bonds. In this version, we have made substantial extensions in order to improve the quality and completeness of our contributions. We have proposed a new method for modeling hydrophobic interactions. We have added an analysis comparing results of our system, KINARI, with published results from an earlier protein rigidity analysis called MSU-FIRST. An additional 9 PDB files have been added to the to the data set in order to compare our system with MSU-FIRST, and additional case studies are presented. The new options for calculating hydrophobics with energies are now available from the KINARI-Web server, as well as a new feature to view histograms of the distribution of interactions by energy. We have made the scripts and dataset for performing the benchmarking available for download from the KINARI website.

## Declaration

Publication of this article was supported by DMS-0714934, NSF CCF-1016988 and DARPA 23 Mathematical Challenges grants of IS.

This article has been published as part of *BMC Bioinformatics *Volume 14 Supplement 18, 2013: Selected articles from the Second IEEE International Conference on Computational Advances in Bio and Medical Sciences (ICCABS 2012): Bioinformatics. The full contents of the supplement are available online at http://www.biomedcentral.com/bmcbioinformatics/supplements/14/S18.

## References

[B1] Jacobs DJ Rader AJ Thorpe MF Kuhn LA Protein Flexibility Predictions using Graph Theory Proteins 2001 44 150 165 10.1002/prot.1081 11391777

[B2] Chubynsky MV Hespenheide BM Jacobs DJ Kuhn LA Lei M Menor S Rader AJ Thorpe MF Whiteley W Zavodszky MI Constraint Theory applied to Proteins Nanotechnology Research Journal 2008 2 61 72

[B3] Fox N Jagodzinski F Li Y Streinu I KINARI-Web: A Server for Protein Rigidity Analysis Nucleic Acids Res 2011 39 Web Server http://kinari.cs.umass.edu 10.1093/nar/gkr482 PMC3125808 21693559

[B4] Bagga A Baldwin B Entity-based cross-document coreferencing using the Vector Space Model Proceedings of the Association for Computational Linguistics (ACL) 1998 79 85

[B5] Jacobs DJ Kuhn LA Thorpe MF Flexible and Rigid Regions in Proteins Rigidity Theory and Applications 1999 Kluwer Academic 357 384

[B6] Abyzov A Bjornson R Felipe M Gerstein M RigidFinder: a fast and sensitive method to detect rigid blocks in large macromolecular complexes Proteins 2010 78 2 309 324 10.1002/prot.22544 19705487

[B7] Hespenheide BM Rader AJ Thorpe MF Kuhn LA Identifying Protein Folding Cores: Observing the Evolution of Rigid and Flexible Regions During Unfolding J Mol Graph Model 2002 21 3 195 20 10.1016/S1093-3263(02)00146-8 12463638

[B8] Thomas S Tang X Tapia L Amato NM Simulating Protein Motions with Rigidity Analysis J Comput Biol 2007 14 6 839 855 10.1089/cmb.2007.R019 17691897

[B9] Wells SA Menor S Hespenheide BM Thorpe MF Constrained Geometric Simulation of Diffusive Motion in Proteins Phys Biol 2005 2 S127 S136 10.1088/1478-3975/2/4/S07 16280618

[B10] Gohlke H Kuhn LA Case DA Change in protein flexibility upon complex formation: analysis of Ras-Raf using molecular dynamics and a molecular framework approach Proteins 2004 56 2 322 337 10.1002/prot.20116 15211515

[B11] Wells SA Jimenez-Roldan JE Romer RA Comparative analysis of rigidity across protein families Phys Biol 2009 6 4 10.1088/1478-3975/6/4/046005 19773604

[B12] Gilli G Gilli P The Nature of the Hydrogen Bond, Outline of a Comprehensive Hydrogen Bond Theory 2009 Oxford University Press

[B13] Jeffrey G Saenger W Hydrogen Bonding in Biological Structures 1991 Springer-Verlag

[B14] Panigrahi S Desiraju G Strong and weak hydrogen bonds in the protein-ligand interface Proteins 2007 67 128 141 10.1002/prot.21253 17206656

[B15] Del Carpio CA Shaikh AR Ichiishi E Koyama M Nishijima K Miyamoto A A graph theoretical approach for analysis of protein flexibility change at protein complex formation Genome Inform 2005 16 2 148 160 16901098

[B16] Petsko GA Ringe D Protein Structure and Function 2004 New Science Press

[B17] Mayo SL Dahiyat BI Gordon DB Automated design of the surface positions of protein helices Protein Sci 1997 6 6 1333 1337 10.1002/pro.5560060622 9194194 PMC2143725

[B18] Kortemme T Morozov AV Baker D An orientation-dependent hydrogen bonding potential improves prediction of specificity and structure for proteins and protein-protein complexes J Mol Biol 2003 326 4 1239 1259 10.1016/S0022-2836(03)00021-4 12589766

[B19] Wang J Cieplak P Kollman PA How well does a restrained electrostatic potential (RESP) model perform in calculating conformational energies of organic and biological molecules? J Comput Chem 2000 21 1049 1074 10.1002/1096-987X(200009)21:12<1049::AID-JCC3>3.0.CO;2-F

[B20] Tay TS Rigidity of multigraphs I: linking rigid bodies in n-space J Combinatorial Theory, Series B 1984 26 95 112

[B21] Tay TS Whiteley W Recent advances in the generic rigidity of structures Structural Topology 1984 9 31 38

[B22] Lee-St John A Streinu I Pebble Game Algorithms and Sparse Graphs Discrete Mathematics 2008 308 8 1425 1437 10.1016/j.disc.2007.07.104

[B23] Katoh N Tanigawa S A Proof of the Molecular Conjecture Discrete and Computational Geometry 2011 45 4 647 700 10.1007/s00454-011-9348-6

[B24] Jacobs DJ Generic rigidity in three-dimensional bond-bending networks J Phys A: Math Gen 31 1998 31 6653 6668 10.1088/0305-4470/31/31/012

[B25] Flexweb: Analysis of Flexibility in Biomolecules and Networks http://flexweb.asu.edu

[B26] Thorpe MF Hespenheide BM Yang Y Kuhn LA Flexibility and critical hydrogen bonds in cytochrome c Pacific Symposium on Biocomputing 2000 191 202 10902168 10.1142/9789814447331_0018

[B27] Keating K Flores S Gerstein M Kuhn LA StoneHinge: Hinge prediction by network analysis of individual protein structures Protein Sci 2008 18 2 359 371 19180449 10.1002/pro.38 PMC2708048

[B28] Rand WM Objective Criteria for the Evaluation of Clustering Methods J Am Stat Assoc 1971 66 336 846 850 10.1080/01621459.1971.10482356

[B29] Gonzalez LC Wang H Livesay DR Jacobs DJ Calculating ensemble averaged descriptions of protein rigidity without sampling PLoS One 2012 7 2 10.1371/journal.pone.0029176 PMC3285152 22383947

[B30] Kurnikova M Mamonova T Hespenheide BM Straub R Protein flexibility using constraints from molecular dynamics simulations Phys Biol 2005 2 4 S137 S147 10.1088/1478-3975/2/4/S08 16280619

[B31] Meila M Comparing clusterings: an axiomatic view Proceedings of the International Conference on Machine Learning (ICML) 2005 577 584

[B32] Gusfield D Partition-distance: A problem and class of perfect graphs arising in clustering Information Processing Letters 2002 82 159 164 10.1016/S0020-0190(01)00263-0

[B33] Weber IT Agniswamy J HIV-1 Protease: Structural Perspectives on Drug Resistance Viruses 2009 1 3 1110 1136 10.3390/v1031110 21994585 PMC3185505

[B34] Fulle S Gohlke H Analyzing the Flexibility of RNA Structures by Constraint Counting Biophysics J 2008 94 4202 4219 10.1529/biophysj.107.113415 PMC2480660 18281388

[B35] Fox N Streinu I Redundant Interactions in Protein Rigid Cluster Analysis1st IEEE International Conference on Computational Advances in Bio and medical Sciences 2011 99 104

[B36] McDonald I Thornton JM Satisfying Hydrogen Bonding Potential in Proteins J Mol Bio 1994 238 5 777 793 10.1006/jmbi.1994.1334 8182748

